# Respiratory Support in Cardiogenic Pulmonary Edema: Clinical Insights from Cardiology and Intensive Care

**DOI:** 10.3390/jcdd13010054

**Published:** 2026-01-20

**Authors:** Nardi Tetaj, Giulia Capecchi, Dorotea Rubino, Giulia Valeria Stazi, Emiliano Cingolani, Antonio Lesci, Andrea Segreti, Francesco Grigioni, Maria Grazia Bocci

**Affiliations:** 1 Cardiology Unit, Campus Bio-Medico University Hospital, Via Alvaro del Portillo 200, 00128 Rome, Italy; nardi.tetaj@unicampus.it (N.T.); a.segreti@policlinicocampus.it (A.S.); f.grigioni@policlinicocampus.it (F.G.); 2 Intensive Care and Anesthesiology Unit, Clinical and Research Department, National Institute for Infectious Diseases INMI Lazzaro Spallanzani, Scientific Institute for Research, Hospitalization and Healthcare (IRCCS), 00149 Rome, Italy; dorotea.rubino@inmi.it (D.R.); giuliavaleria.stazi@inmi.it (G.V.S.); mariagrazia.bocci@inmi.it (M.G.B.); 3 Intensive Care for Trauma and Shock General Hospital, San Camillo Forlanini, 00149 Rome, Italy; ecingolani@scamilloforlanini.rm.it; 4 Istituto di Anestesiologia e Rianimazione, Università Cattolica del Sacro Cuore, 00136 Rome, Italy; antonio.lesci01@icatt.it

**Keywords:** cardiogenic pulmonary edema, acute respiratory failure, respiratory support, noninvasive ventilation, continuous positive airway pressure, bilevel positive airway pressure

## Abstract

Cardiogenic pulmonary edema (CPE) is a life-threatening manifestation of acute heart failure characterized by rapid accumulation of fluid in the interstitial and alveolar spaces, leading to severe dyspnea, hypoxemia, and respiratory failure. The condition arises from elevated left-sided filling pressures that increase pulmonary capillary hydrostatic pressure, disrupt alveolo-capillary barrier integrity, and impair gas exchange. Neurohormonal activation further perpetuates congestion and increases myocardial workload, creating a vicious cycle of hemodynamic overload and respiratory compromise. Respiratory support is a cornerstone of management in CPE, aimed at stabilizing oxygenation, reducing the work of breathing, and facilitating ventricular unloading while definitive therapies, such as diuretics, vasodilators, inotropes, or mechanical circulatory support (MCS), address the underlying cause. Among available modalities, non-invasive ventilation (NIV) with continuous positive airway pressure (CPAP) or bilevel positive airway pressure (BiPAP) has the strongest evidence base in moderate-to-severe CPE, consistently reducing the need for intubation and providing rapid relief of dyspnea. High-flow nasal cannula (HFNC) represents an emerging alternative in patients with moderate hypoxemia or intolerance to mask ventilation, and should be considered an adjunctive option in selected patients with less severe disease or NIV intolerance, although its efficacy in severe presentations remains uncertain. Invasive mechanical ventilation is reserved for refractory cases, while extracorporeal membrane oxygenation (ECMO) and other advanced circulatory support modalities may be necessary in cardiogenic shock. Integration of respiratory strategies with hemodynamic optimization is essential, as positive pressure ventilation favorably modulates preload and afterload, synergizing with pharmacological unloading. Future directions include personalization of ventilatory strategies using advanced monitoring, novel interfaces to improve tolerability, and earlier integration of MCS. In summary, respiratory support in CPE is both a bridge and a decisive therapeutic intervention, interrupting the cycle of hypoxemia and hemodynamic deterioration. A multidisciplinary, individualized approach remains central to improving outcomes in this high-risk population.

## 1. Introduction

Cardiogenic pulmonary edema (CPE) represents one of the most dramatic and life-threatening manifestations of acute heart failure. It results from a sudden rise in left-sided filling pressures, leading to fluid transudation into the pulmonary interstitium and alveoli, and culminates in acute respiratory distress and hypoxemia [1]. Despite advances in pharmacological and device-based therapies, CPE continues to be associated with substantial morbidity, frequent intensive care admissions, and significant in-hospital mortality [2,3].

The clinical burden of CPE is considerable. In contemporary cohorts of acute heart failure, up to one-third of hospitalizations are complicated by pulmonary edema, with many patients requiring respiratory support within hours of presentation. Beyond its immediate hemodynamic implications, the development of alveolar flooding profoundly impairs gas exchange, increases the work of breathing, and triggers neurohormonal activation, thereby exacerbating left ventricular dysfunction in a vicious cycle [4,5].

Respiratory support constitutes a cornerstone of management in CPE, complementing diuretics, vasodilators, and inotropes [6]. Over the past three decades, non-invasive ventilation (NIV) has emerged as the preferred modality, supported by robust evidence demonstrating rapid improvement in oxygenation, relief of dyspnea, and reduced need for intubation [7]. More recently, high-flow nasal cannula (HFNC) and refined invasive ventilation strategies have expanded the armamentarium, while mechanical circulatory support (MCS) may be required in cases complicated by cardiogenic shock [8,9].

Given the evolving landscape, a comprehensive understanding of the physiopathology of CPE and the rationale for respiratory interventions is essential for optimizing clinical outcomes. This review evaluates the mechanisms underlying CPE, summarizes current evidence for different respiratory support modalities, and discusses their integration with hemodynamic management in critically ill patients.

### Search Strategy and Selection Criteria

This narrative review was based on a targeted literature search of PubMed/MEDLINE and Embase covering the period from 2000 to September 2025. The search strategy used the terms cardiogenic pulmonary edema and acute respiratory failure, with key terms related to respiratory support, HFNC, NIV, continuous positive airway pressure (CPAP), bilevel positive airway pressure, mechanical circulatory support, cardiogenic shock, extracorporeal membrane oxygenation (ECMO), Impella, and left ventricular unloading. We excluded proceeding papers, corrections, early access articles, news items, book chapters, retractions, reprints, biographical items, book reviews, meeting abstracts, editorial materials, and letters. We included relevant English-language studies involving adult populations, with particular focus on randomized controlled trials and large observational studies. Given the narrative design, study selection prioritized clinical relevance, methodological quality, and applicability to contemporary practice.

## 2. Physiopathology of Cardiogenic Pulmonary Edema

CPE arises from elevated left-sided cardiac filling pressures, most commonly due to left ventricular (LV) systolic or diastolic dysfunction, acute myocardial infarction, valvular disease, or arrhythmias [1]. The pathophysiological cascade can be understood as the interplay between hemodynamic status, alveolo-capillary membrane integrity, and neurohormonal activation. The initiating event is impaired LV performance, which leads to a rise in LV end-diastolic pressure (LVEDP). This pressure is transmitted retrogradely to the left atrium and pulmonary veins, resulting in elevated pulmonary capillary wedge pressure (PCWP). Under physiological conditions, PCWP is approximately 8–12 mmHg; when it exceeds the plasma oncotic pressure (25 mmHg), alveolar capillary integrity may be compromised, leading to increased permeability. Consequently, fluid transudation into the pulmonary interstitium exceeds the lymphatic interstitial drainage capacity, leading initially to interstitial edema and, at higher pressures, to alveolar flooding [10,11]. Fluid accumulation within alveoli not only impairs oxygen diffusion but also dilutes and inactivates pulmonary surfactant. The loss of surfactant activity promotes alveolar collapse, reduces lung compliance, and increases the work of breathing [12].

The hemodynamic derangements of CPE are compounded by sympathetic nervous system overactivity, which increases heart rate, systemic vascular resistance through vasoconstriction, and myocardial oxygen demand. Vasoconstriction of the postcapillary pulmonary veins raises PCWP, while concomitant systemic arterial vasoconstriction redistributes blood volume toward the pulmonary circulation. Activation of the renin–angiotensin–aldosterone system (RAAS) promotes sodium and water retention, perpetuating congestion, thereby establishing a vicious cycle of escalating pulmonary edema [13,14].

Beyond hemodynamics, sympathetic hyperactivity and excessive catecholamine release trigger an abnormal inflammatory response [15]. RAAS activation further amplifies inflammatory pathways [16]. Together, these mechanisms stimulate cytokine release, increase pulmonary capillary permeability, and aggravate alveolar fluid accumulation, reinforcing the spiral of inflammation, vascular leak, and congestion [17].

Right ventricular (RV) involvement plays a pivotal role. Sustained elevation of left atrial and pulmonary venous pressures is transmitted to the pulmonary circulation, leading to pulmonary hypertension and increased RV afterload. The RV, being thin-walled and poorly adapted to acute pressure overload, dilates and develops elevated right-sided filling pressures. This backward transmission into the systemic venous circulation produces peripheral edema, hepatic engorgement, hepatomegaly, and jugular venous distension [18]. RV dilation also exacerbates ventricular interdependence by shifting the interventricular septum toward the LV cavity and further impairing LV filling and output. The coexistence of pulmonary and systemic venous congestion thus defines the progression to biventricular failure in CPE [19,20].

The progression from interstitial to alveolar edema profoundly disrupts pulmonary gas exchange. Hypoxemia arises from multiple mechanisms. Some regions are perfused but poorly ventilated due to alveolar fluid accumulation, leading to a ventilation–perfusion (V/Q) mismatch and intrapulmonary right-to-left shunting through flooded alveoli. These mechanisms explain hypoxemia, which can be severe and refractory to oxygen therapy [21]. Pulmonary interstitial edema also stimulates juxtacapillary (J) receptors, triggering tachypnea and the sensation of dyspnea [22].

Although arterial partial pressure of carbon dioxide (PaCO_2_) is low initially due to hyperventilation, in advanced stages, progressive fatigue or severe shunt impairs CO_2_ clearance, contributing to hypercapnic respiratory failure [23].

**Figure 1:**
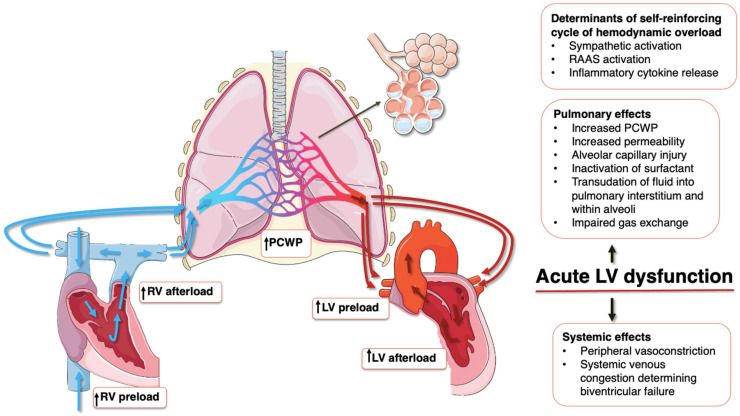
Pathophysiological mechanisms of CPE. Schematic illustration showing how left ventricular dysfunction results in hemodynamic overload transmitted retrogradely, leading to increased left atrial pressure, pulmonary venous congestion, alveolar-capillary membrane disruption, and alveolar flooding. Neurohormonal activation, inflammation, and elevated RV filling pressure further contribute to a self-reinforcing cycle of worsening hemodynamic overload and thus pulmonary edema. Abbreviations: LV, left ventricle; RV, right ventricle; PCWP, pulmonary capillary wedge pressure; RAAS, renin–angiotensin–aldosterone system; blue arrows indicate the flow of oxygen-poor blood; red arrow indicate the flow of oxygen-rich blood.

In summary, CPE represents a self-reinforcing cycle of hemodynamic overload, neurohormonal and inflammatory activation, RV dysfunction, and impaired gas exchange (Figure 1). This cascade underscores the urgency of timely recognition and intervention to prevent rapid clinical deterioration.

## 3. Diagnostic Approach to Cardiogenic Pulmonary Edema

Early diagnosis of CPE is essential, as timely respiratory and hemodynamic stabilization is often effective. Patients typically present with acute dyspnea, orthopnea, tachypnea, and signs of respiratory distress, frequently in the setting of known heart failure, ischemic heart disease, or hypertension [1]. Clinical severity should be promptly assessed, as marked work of breathing, persistent hypoxemia despite supplemental oxygen, altered mental status, or hypotension identify patients requiring early escalation of respiratory and circulatory support. Arterial blood gas analysis usually reveals hypoxemia due to intrapulmonary shunt, while hypercapnia, when present, suggests advanced disease or respiratory muscle fatigue and predicts NIV failure [24,25]. Laboratory testing should support a cardiogenic mechanism and identify precipitants, including natriuretic peptides, cardiac troponin, lactate, renal function, and electrolytes [26,27]. Chest X-ray may demonstrate vascular redistribution, interstitial or alveolar edema, cardiomegaly, and pleural effusions, although radiographic findings can be absent early and lag behind clinical improvement [28]. Early focused echocardiography is crucial to confirm cardiac involvement, assess left ventricular systolic and diastolic function, identify acute valvular disease or mechanical complications, evaluate right ventricular function, and stratify hemodynamic severity, particularly in patients with hypotension or suspected cardiogenic shock [29]. Bedside lung ultrasound following the Lichtenstein approach represents a first-line diagnostic tool for CPE, allowing rapid differentiation between hydrostatic and non-cardiogenic causes of pulmonary infiltrates [30]. A diffuse, bilateral B-profile with smooth pleural lines strongly supports a cardiogenic mechanism and should prompt immediate integration with focused cardiac ultrasound to confirm left-sided cardiac dysfunction, identify precipitating factors, and guide early therapeutic decisions [31], Table 1.

## 4. Rationale for Respiratory Support

CPE is one of the most common causes of acute respiratory failure in the acute care setting and often requires respiratory support. Clinically, CPE manifests as sudden-onset dyspnea, orthopnea, and diffuse pulmonary rales. In severe cases, frothy and occasionally blood-tinged sputum reflects severe alveolar edema. Without timely intervention, the combination of worsening gas exchange, escalating work of breathing, and hemodynamic compromise leads to respiratory failure and progression to cardiogenic shock in hours or minutes [32]. Prompt recognition and early treatment are therefore critical to prevent rapid clinical deterioration.

Patients with CPE should be closely monitored for signs of worsening respiratory status and decreased gas exchange, and they typically require stepwise escalation of respiratory support. The objective of respiratory support in CPE is to improve oxygenation, reduce the work of breathing, and buy time for definitive hemodynamic interventions [10]. According to the ESC guidelines in the setting of acute heart failure, oxygen is recommended in patients with SpO_2_ < 90% or PaO_2_ < 60 mmHg to correct hypoxemia (CoR I, LOE C) [33]. As a first step, conventional oxygen therapy (COT), which includes delivering nasal cannulas or a face mask (Venturi or reservoir mask), is the preferred initial approach.

A nasal cannula is the most common oxygen delivery system, typically indicated for patients with mild hypoxemia. It delivers oxygen into the nasopharyngeal space at flow rates of 1–6 L/min, corresponding to FiO_2_ of approximately 24–40%, respectively. The reservoir mask has a bag that collects high concentrations of oxygen (close to 100%), and one-way valves prevent exhaled air from re-entering the bag. The Venturi mask mixes oxygen with room air using the Venturi effect, delivering a fixed and accurate FiO_2_ (typically 24% to 60%), making them useful in patients where minimizing carbon dioxide retention is essential, such as COPD [34]. COT is simple to use, comfortable and tolerable for the patient, and does not require expertise. However, COT is often insufficient, necessitating stepwise escalation to high-flow nasal oxygen (HFNO), CPAP, or NIV, most commonly “bilevel” positive airway pressure (bilevel NIV or BiPAP), according to the patient’s clinical status. During oxygen therapy, acid-base balance and SpO_2_ should be monitored [35].

HFNO delivers heated (31–37 °C) and humidified oxygen at controlled concentrations and high flow rates (up to 60–80 L/min) through a nasal cannula. It is generally comfortable, well tolerated, and allows patients with acute respiratory failure to speak, eat and drink. HFNO also generates a mild level of positive end-expiratory pressure (PEEP); with a closed mouth and a flow of 60 L/min, PEEP may reach approximately 7 cmH_2_O. However, this effect is easily attenuated when the mouth is opened. Also, it helps wash out CO_2_ by reducing dead space in the upper airway [36,37,38].

Although HFNC may improve comfort and oxygenation in selected patients with mild-to-moderate CPE, current evidence does not support its use as a substitute for NIV in severe presentations. In patients with marked respiratory distress, significant hypoxemia, CPAP or bilevel NIV remains the preferred first-line strategy, given its superior ability to reduce preload and afterload and its stronger evidence base [39].

When respiratory distress persists (respiratory rate > 25 breaths/min, SpO_2_ < 90%), despite COT or HFNO, the ESC guidelines recommend considering CPAP or bilevel NIV as early as possible, given its superior ability to reduce preload and afterload, decrease respiratory distress, reduce the need for endotracheal intubation and its stronger evidence base (CoR IIa, LOE B) [33,40]. Similarly, the Official ERS/ATS clinical practice guidelines on NIV for acute respiratory failure recommend either bilevel NIV or CPAP for patients with ARF due to CPE, with a strong recommendation and moderate certainty of evidence [41]. In patients with ARF due to CPE, the application of positive airway pressure improves oxygenation by recruiting alveoli, reducing intrapulmonary shunt and improving V/Q mismatch. In addition, it decreases systemic venous return and LV afterload, thereby reducing LV filling, lowering pulmonary hemodynamic overload, favouring myocardial contraction and alleviating congestion. Reducing the work of breathing also prevents respiratory muscle fatigue [42].

**Figure 2:**
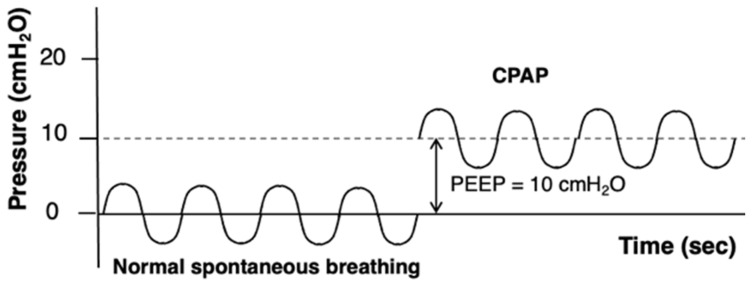
Example of pressure/time curves in continuous positive airway pressure. Abbreviations: CPAP, continuous positive airway pressure; PEEP, positive end-expiratory pressure.

CPAP delivers a constant preset positive pressure throughout the entire respiratory cycle, Figure 2. Compared with bilevel NIV, it offers the possibility of application without a mechanical ventilator, the advantages of simpler technology, and more effortless patient–device synchronization [43,44]. CPAP requires an effective spontaneous respiratory drive, as it delivers continuous pressure without providing inspiratory support to augment tidal volume. CPAP can be delivered via an oronasal mask, a full-face mask, or a helmet. It is usually initiated at higher levels (up to 12.5 cmH_2_O) reserved for selected patients [45,46,47]. Close monitoring of symptoms, respiratory rate, and hemodynamic stability is essential, and lack of improvement or clinical deterioration should prompt escalation to bilevel NIV or invasive mechanical ventilation.

**Figure 3:**
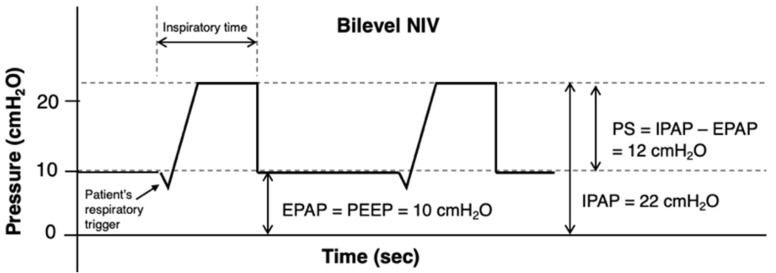
Example of pressure/time curves in bilevel noninvasive ventilation. The arrow indicates the patient’s inspiratory trigger initiating ventilator flow delivery to achieve the preset PS. Abbreviations: PEEP, positive end-expiratory pressure; NIV, noninvasive ventilation; IPAP, inspiratory positive airway pressure; EPAP, expiratory positive airway pressure; PS, pressure support.

In contrast, bilevel NIV supplies additional inspiratory pressure, as shown in Figure 3, thereby reducing the work of breathing and facilitating carbon dioxide clearance. It typically delivers a positive inspiratory pressure above the set baseline PEEP level with each patient-triggered breath, increasing minute ventilation and reducing respiratory rate. It should also be considered in patients with hypoxic hypercapnic respiratory failure and remains the preferred modality for those with COPD who develop acute respiratory acidosis during hospitalization, as it effectively reverses both hypercapnia and acidosis [48,49,50]. Hemodynamic status should also guide modality selection: in hypertensive “flash” pulmonary edema, NIV is generally well tolerated and synergizes with vasodilator therapy, whereas in hypotensive patients or those with evolving cardiogenic shock, NIV should be applied cautiously with lower initial pressures and close monitoring, with early escalation to invasive ventilation or MCS if instability persists [51].

Bilevel NIV is usually initiated with an inspiratory positive airway pressure (IPAP) of 6–12 cmH_2_O and an expiratory positive airway pressure (EPAP) of 4–8 cmH_2_O, titrated according to gas exchange and patient tolerance. IPAP may be increased in 2–3 cmH_2_O increments (up to 20–25 cmH_2_O) to improve tidal volume (6–8 mL/kg) and facilitate CO_2_ clearance. At the same time, EPAP, which is equivalent to PEEP, is adjusted to optimize oxygenation and maintain alveolar recruitment. The FiO_2_ is titrated to achieve target saturations (≥92–94%, or 88–92% in patients at risk of hypercapnia) [52,53]. The difference between IPAP and EPAP represents the pressure support (PS), which determines ventilatory assistance. A backup rate can be set in cases of hypercapnia. The maximum peak inspiratory pressure should be kept ≤30 cmH_2_O to minimize the risk of pulmonary barotrauma [54]. The choice of interface (oronasal or full-face) for individual patients is critical to determining NIV success or failure, since poor tolerance is often associated with the interface [55]. In patients with RV dysfunction, hypovolemia or severe hypotension, excessive positive pressure may critically reduce preload and cardiac output. Hence, careful titration is mandatory. Furthermore, in addition to hemodynamics, cautious monitoring of respiratory rate, comfort, and arterial blood gases within 30–60 min is essential; failure to improve should prompt consideration of intubation.

The primary limitation of delivering NIV via a face mask is the occurrence of significant air leaks, which result from an inadequate seal between the mask and the patient’s face [56]. Prolonged use of oronasal masks is frequently associated with skin breakdown, particularly over the nasal bridge. To improve comfort and tolerance, alternating between oronasal and full-face masks may be beneficial. Additional complications include gastric insufflation and impaired secretion clearance, which often necessitate treatment interruptions. These challenges are especially problematic during prolonged NIV, thereby limiting its effectiveness in this setting [57,58].

Sedation and analgesia may be employed during NIV to improve patient comfort and tolerance, particularly in agitated or anxious patients. The most commonly used agents include short-acting benzodiazepines, dexmedetomidine, and low-dose intravenous opioids, typically administered in small boluses. Dexmedetomidine provides anxiolysis and light sedation with minimal respiratory depression, although it may cause bradycardia and hypotension. By contrast, benzodiazepines and opioids may reduce ventilatory drive and therefore should be used cautiously. Regardless of the agent, continuous monitoring of consciousness, oxygenation, and ventilation is essential to avoid hypoventilation and to promptly recognize NIV failure [59,60].

In 2008, Gray et al. [61], published the largest multicenter trial, from 26 emergency departments, in which 1069 patients with acute CPE were randomized to CPAP, bilevel NIV or standard oxygen therapy. This trial found rapid improvement in respiratory distress and metabolic disturbances in the CPAP and bilevel NIV groups compared with the standard group, but no difference in intubation rate or short-term mortality. However, interpretation of the results was limited by the high crossover rate in the oxygen group that crossed over to bilevel NIV [61]. Subsequently, systematic reviews have incorporated the data from Gray et al. [61], as well as other subsequent trials, and consistently concluded that CPAP and bilevel NIV decrease the need for intubation and in-hospital mortality, findings that are in line with the ERS/ATS guideline for NIV in acute respiratory failure [62,63]. Invasive mechanical ventilation should be considered in patients with CPE edema who exhibit persistent hypoxemia despite optimal NIV, severe respiratory acidosis (pH < 7.25 with rising PaCO_2_), respiratory exhaustion, altered mental status, inability to protect the airway, or worsening hemodynamic instability. In such cases, early intubation may prevent catastrophic deterioration and should be integrated with aggressive hemodynamic optimization [64,65].

Table 2 summarizes the different types of respiratory support with O_2_ therapy, reporting indications, advantages, limits, contraindications and complications.

## 5. Hemodynamic Management

To improve conceptual clarity, respiratory support should be viewed as an integral component of hemodynamic management rather than an isolated intervention. Positive pressure ventilation favorably interacts with pharmacological therapy by reducing preload and left ventricular afterload, thereby enhancing the effects of diuretics and vasodilators on pulmonary congestion. In parallel, improved oxygenation and reduced work of breathing lower sympathetic activation and myocardial oxygen demand. In patients with cardiogenic shock, ventilatory support complements inotropes, vasopressors, and MCS by stabilizing gas exchange while definitive unloading strategies are implemented [5,10]. Table 3 summarizes the complementary physiological effects of respiratory support, pharmacological therapy, and MCS across different hemodynamic profiles, providing a unified framework for integrated cardio–respiratory management.

Loop diuretics remain the first-line pharmacologic therapy for acute CPE, promoting rapid intravascular volume reduction. NIV enhances their effect by further reducing preload, accelerating the resolution of congestion [66]. Agents such as nitrates are rapidly acting and reduce preload and afterload. In patients with hypertensive pulmonary edema, the combination of vasodilators and NIV is particularly effective, often producing dramatic symptomatic improvement within minutes. However, nitrates should not be used in hypotensive patients (blood pressure < 110 mmHg), and they should be used with extreme caution in patients with aortic stenosis [67].

The use of morphine sulfate in CPE has long been standard practice. However, robust evidence supporting a beneficial hemodynamic effect is lacking [68]. Its perceived benefit may be primarily attributable to anxiolysis, leading to reduced catecholamine release and a consequent decrease in systemic vascular resistance. Conversely, data suggest that morphine may be associated with worse outcomes, such as an increased need for ICU admission and endotracheal intubation [69]. In patients with severe anxiety, low-dose benzodiazepines may represent a safer alternative. Importantly, adverse effects such as nausea, vomiting, and respiratory depression occur more frequently with morphine than with benzodiazepines, which may outweigh any potential benefit [70].

When CPE is complicated by cardiogenic shock, pharmacological hemodynamic support is often required. Inotropes such as dobutamine, milrinone, or levosimendan enhance contractility and cardiac output but at the expense of increased myocardial oxygen demand and a heightened risk of arrhythmias [71]. Vasopressors, most commonly norepinephrine, are indicated to maintain perfusion pressure in profound hypotension; however, they increase afterload and may exacerbate pulmonary congestion. Careful balance of ventilatory strategies, such as NIV, can reduce afterload and promote forward flow, whereas excessive vasopressor use may counteract these hemodynamic benefits [72].

As a rescue therapy, when pharmacological strategies prove insufficient, MCS can provide both hemodynamic stabilization and decongestion. Available devices include the intra-aortic balloon pump (IABP), Impella and veno-arterial ECMO (VA-ECMO).

In this context, early LV unloading with percutaneous devices such as Impella provides a direct reduction in LV end-diastolic pressure and left atrial pressure, thereby addressing the primary hemodynamic driver of CPE. Recent randomized evidence has demonstrated that early Impella-based LV unloading in acute myocardial infarction–related cardiogenic shock is associated with improved survival, underscoring the prognostic importance of timely mechanical support [73]. From a physiological standpoint, LV unloading synergizes with positive pressure ventilation by further reducing pulmonary capillary hydrostatic pressure, facilitating alveolar decongestion, and improving lung compliance, which may in turn enhance tolerance of NIV and reduce the need for invasive mechanical ventilation. These data support an integrated cardio–respiratory strategy in severe presentations, where early combination of respiratory support and LV unloading may interrupt the vicious cycle of hypoxemia, elevated filling pressures, and ongoing myocardial stress [74].

**Figure 4:**
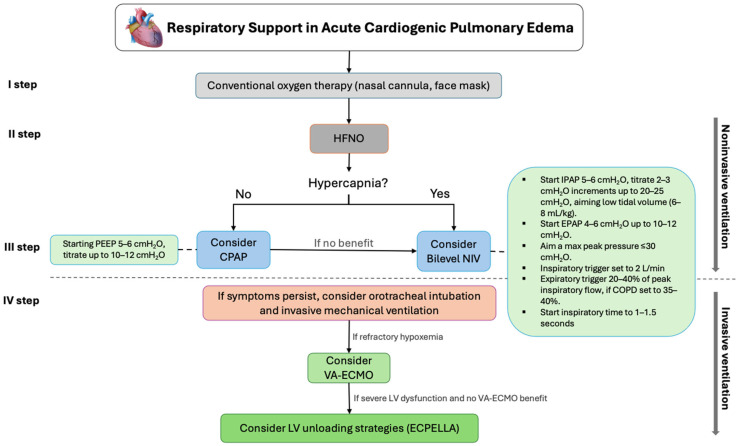
Example of an algorithm (authors’ viewpoint) for the initial management of patients with acute respiratory failure due to CPE Abbreviations: CPAP, continuous positive airway pressure; PEEP, positive end-expiratory pressure; NIV, noninvasive ventilation; IPAP, inspiratory positive airway pressure; EPAP, expiratory positive airway pressure; VA-ECMO, veno-arterial extracorporeal membrane oxygenation; LV, left ventricular; ECPELLA, combination of VA-ECMO and Impella.

VA-ECMO provides full systemic support, providing both circulatory and respiratory assistance in patients with severe, refractory acute respiratory failure due to CPE and cardiogenic shock, as shown in Figure 4. However, careful patient selection is essential, as VA-ECMO increases LV afterload, left atrial and pulmonary venous pressures, and may exacerbate pulmonary congestion [75,76]. To counteract these complications, LV unloading strategies are frequently employed, most commonly with the Impella device or, less frequently, the IABP. The combined use of VA-ECMO and Impella, termed “ECPELLA”, enables adequate systemic support while simultaneously unloading the LV [77,78]. Although randomized controlled trial (RCT) data remain limited, observational studies suggest that LV unloading during VA-ECMO may confer a survival advantage. Nonetheless, potential complications are not uncommon, including bleeding, limb ischemia, LV distension, and pulmonary edema progression, underscoring the importance of experienced multidisciplinary management [79,80].

## 6. Conclusions

CPE epitomizes the interplay between impaired cardiac function and acute respiratory failure. Its rapid onset and high mortality risk demand timely recognition and prompt initiation of supportive strategies. Respiratory support is pivotal in this setting, not only as a means of stabilizing gas exchange but also as a therapeutic intervention that favorably modifies cardiac loading conditions.

Among available modalities, CPAP and Bilevel NIV remains the cornerstone of therapy, consistently reducing the need for intubation and providing immediate relief of dyspnea. HFNC may be considered in less severe cases or when NIV is poorly tolerated. At the same time, invasive ventilation and ECMO and/or Impella are reserved for refractory hypoxemia or advanced cardiogenic shock. Importantly, respiratory interventions achieve maximal benefit when integrated with hemodynamic optimization through diuretics, vasodilators, inotropes, and, in selected patients, MCS.

Despite robust evidence for NIV, uncertainties remain regarding patient stratification, the role of HFNC in severe CPE, and the optimal timing of advanced mechanical support. Future research should prioritize personalized approaches that leverage physiological monitoring, novel ventilatory interfaces, and early integration of circulatory support strategies.

In summary, respiratory support in CPE is both a bridge to definitive therapy and a decisive intervention capable of interrupting the vicious cycle of hypoxemia and hemodynamic compromise. A multidisciplinary, individualized approach remains essential to improve outcomes in this critically ill population. Tailoring the strategy according to the severity of the shock ensures the best outcomes.

## Figures and Tables

**Table 1:** Differential diagnosis of different types of pulmonary edema by lung ultrasound.

	Cardiogenic Pulmonary Edema	ALI/ ARDS	Chronic Heart Failure	Pulmonary Fibrosis	COPD/ Emphysema
Clinical setting	Acute	Acute	Chronic	Chronic	Chronic (often acute exacerbation)
B-lines no.	++++	++++	+/++/+++	+/++/+++	Absent or rare
B-lines distribution	Multiple, diffuse, bilateral, “Wet lung” pattern	Non-homogeneous distribution, spared areas	Multiple, diffuse, bilateral, gravity-dependent	More frequently posterior or lung bases	Absent; predominant A-lines
Other LUS signs	Pleural effusion; smooth and hyperechoic pleural line	Pleural effusion, irregular pleural line, subpleural parenchymal consolidations of various sizes	Pleural effusions, dynamic changes with decongestion	Pleural thickening	Lung hyperinflation signs, lung sliding preserved; bullae may reduce sliding locally.
Echocardiogram	LV systolic and/or diastolic dysfunction; elevated pulmonary pressures	Cardiac structure and function usually preserved	Chronic LV systolic and/or diastolic dysfunction;	Likely normal LV function	Likely normal LV function

Abbreviations: LUS, lung ultrasound; ALI, acute lung injury; ARDS, acute respiratory distress syndrome; COPD, chronic obstructive pulmonary disease; LV, left ventricular; +, ++, +++, and ++++ indicate increasing numbers of B-lines per screen on lung ultrasound.

**Table 2:** Comparison of different types of respiratory support with O_2_ therapy.

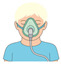 COT	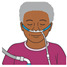 HFNO	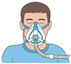 CPAP	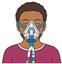 Bilevel NIV
**Indications**			
Hypoxia	Hypoxia	Hypoxia	Hypoxia hypercapnia
Advantages			
Simple use	Simple use	Simple use	Provides inspiratory support
Comfort	Comfort	Provides PEEP	Provides PEEP
No ventilator needed	No ventilator needed	With or without ventilator	Need for ventilatory support
	Low level of PEEP		
	Upper airway washout		
Limits			
No inspiratory support	No inspiratory support	No inspiratory support	Ventilator needed
			Expertise needed
Main Contraindications			
None specific	Epistaxis	GCS ≤ 8	GCS ≤ 8
	Nasal obstruction	Active and persistent vomiting	Active and persistent vomiting
		Severe active upper gastrointestinal bleeding	Severe active upper gastrointestinal bleeding
		Facial or upper airway trauma/burns	Facial or upper airway trauma/burns
		Uncooperative patient	Uncooperative patient
		Facial anatomic anomalies	Facial anatomic anomalies
		Impaired airway protective reflexes	Impaired airway protective reflexes
		Undrained pneumothorax	Undrained pneumothorax
Complications			
Dry nose	Dry nose	Discomfort	Discomfort
	Epistaxis	Claustrophobia	Claustrophobia
		Facial skin lesions	Facial skin lesions
		Aerophagia	Aerophagia
		Air leak	Air leak
			Ventilator asynchrony
			Barotrauma

Abbreviations: COT, conventional oxygen therapy; HFNO, high-flow nasal cannula oxygen; CPAP, continuous positive airway pressure; Bilevel NIV, bilevel noninvasive ventilation; PEEP, positive end-expiratory pressure; GCS, Glasgow Coma Scale.

**Table 3:** Complementary effects of respiratory and hemodynamic therapies in CPE.

Intervention	Primary Respiratory Effect	Primary Hemodynamic Effect
CPAP/Bilevel NIV	Improves oxygenation and reduces work of breathing	Reduces preload and LV afterload by increasing intrapulmonary pressure.
Diuretics	Indirectly reduces pulmonary congestion	Reduces intravascular volume
Vasodilators (Nitroglycerin, nitroprusside)	Reduce pulmonary capillary pressure	Reduce preload by dilating veins and LV afterload by dilating arteries and decreasing peripheral resistance; synergistic with NIV
Inotropes/MCS	Indirect improvement in oxygen delivery	Increases cardiac output and promotes ventricular unloading

Abbreviations: CPAP, continuous positive airway pressure; Bilevel NIV, bilevel noninvasive ventilation; MCS, mechanical circulatory support; LV, left ventricular.

## Data Availability

No new data were created or analyzed in this study.
